# Clarification and reprogramming of glucose and xylose metabolism in *Sphingomonas sanxanigenens* for efficient sanxan biosynthesis

**DOI:** 10.1002/mlf2.70110

**Published:** 2026-10-21

**Authors:** Yue Ming, Zhuangzhuang Shi, Yiting Lin, Jue Li, Weilong Wang, Wei Sha, Guoqiang Li, Mengmeng Wu, Ting Ma

**Affiliations:** ^1^ Key Laboratory of Molecular Microbiology and Technology, Ministry of Education, College of Life Sciences Nankai University Tianjin China; ^2^ School of Biotechnology and Food Science Tianjin University of Commerce Tianjin China

**Keywords:** co‐utilization, lignocellulosic hydrolysate, sanxan, synthesis

## Abstract

Currently, the production of high‐value chemicals from natural lignocellulose is of great significance for a circular economy. In this study, a robust platform was developed based on *Sphingomonas sanxanigenens* NXdP, which co‐utilizes glucose and xylose to synthesize polysaccharide sanxan, for efficient bioconversion of lignocellulose‐derived sugars. Sanxan is a promising high‐value product due to its high viscosity at low concentrations, excellent acid‐gelling capabilities, and unique rheological characteristics. Through a cell growth‐based screening strategy and gene manipulation, a key transporter (0233) required for the uptake of both glucose and xylose was identified, clarifying the co‐utilization mechanism. Additionally, the metabolic pathways of glucose and xylose were individually blocked to assess their contributions and activities. Furthermore, the metabolic network of strain NXdP was reprogrammed via a “flux‐redirection” strategy to develop a potential microbial host with improved sanxan production on mixed sugars. The final engineered strain S09 achieved a sanxan titer of 20.25 ± 0.78 g/kg under mixed‐sugar fermentation, representing a 13.51% ± 4.38% increase compared with the original strain NXdP. The mechanism underlying this enhanced production was revealed by central carbon metabolism (CCM) analysis. Overall, this study provides insights into the efficient conversion of lignocellulose‐derived sugars and offers genetic components for the green production of other high‐value‐added products.

## Impact statement

Lignocellulose, Earth's most abundant renewable carbon source, is underutilized due to microbial carbon catabolite repression (CCR). *Sphingomonas sanxanigenens* NXdP uniquely co‐utilizes glucose and xylose for sanxan synthesis without CCR. We clarified its co‐utilization mechanism by identifying a key co‐transporter and elucidating the metabolic pathways involved. The metabolic network of strain NXdP was reprogrammed via a “flux‐redirection” strategy to enhance sanxan production. Our findings not only provide insights into overcoming CCR but also offer valuable tools (e.g., sugar transporters and metabolic pathways) for the green production of other high‐value products.

## INTRODUCTION

The production of bulk chemicals from renewable bioresources via environmentally friendly bioprocesses is essential for carbon neutrality and a sustainable bioeconomy^1^. Lignocellulose, the most abundant renewable resource on Earth, consists of cellulose (40%–50%), hemicellulose (25%–30%), and lignin (15%–20%)^2^. Following specific pretreatment and enzymatic saccharification, cellulose is depolymerized into glucose, whereas hemicellulose yields xylose as its predominant sugar^3^. The glucose/xylose ratios in hydrolysates from various lignocellulosic feedstocks ranged widely from 0.08 to 6.50^4^. These fermentable sugars can significantly reduce production costs^3^ and mitigate issues with excessive fossil fuel consumption and environmental deterioration^5^. However, most microorganisms show carbon catabolite repression (CCR), a regulatory mechanism that prioritizes glucose utilization over other sugars such as xylose, leading to sequential rather than simultaneous sugar consumption^6^. This glucose‐mediated repression of xylose utilization results in incomplete substrate conversion, prolonged fermentation, and reduced overall productivity^7^. Therefore, simultaneous glucose and xylose utilization is critical for maximizing the efficiency of lignocellulose bioconversion into value‐added products^8^. Over the past decades, numerous strategies have been developed to enable parallel carbon source utilization by eliminating CCR^9^. One successful approach is deleting the phosphotransferase system (PTS) combined with adaptive evolution of host strains. However, PTS knockout often causes growth deficiencies, and extensive manipulations are usually required to restore normal cell growth^10^. Consequently, there is an urgent need to explore robust platform microorganisms that can naturally co‐utilize the monosaccharides derived from lignocellulose hydrolysis^5^.

Exopolysaccharides (EPS) are high‐molecular‐weight carbohydrate polymers secreted by microorganisms into their extracellular environment during physiological life cycles^11^. Due to their unique physicochemical properties, such as nontoxicity, high viscosity, biocompatibility, and biodegradability^12^, EPS are widely applied in the food, pharmaceutical, cosmeceutical, textile, and paper industries^13^. Among these, sphingans (e.g., gellan, welan, and diutan gum) produced by *Sphingomonas* species have attracted significant interest for their remarkable characteristics and broad applications. Most sphingans share a conserved backbone structure: → 4)‐α‐L‐Rha or α‐L‐Man‐(1 → 3)‐β‐D‐Glc‐(1 → 4)‐β‐D‐GlcA‐(1 → 4)‐β‐D‐Glc‐(1 →, with side‐chain variations conferring distinct rheological properties^14^. Unlike typical sphingans, sanxan gum produced by *Sphingomonas sanxanigenens* NXdP (CGMCC 15406) has a tetrasaccharide repeat unit: → 4)‐β‐D‐Man‐(1 → 4)‐β‐D‐GlcA‐(1 → 3)‐α‐L‐Rha‐(1 → 3)‐β‐D‐Glc‐(→1^15^, ^16^. This unique structure endows sanxan with excellent properties, including high viscosity at low concentrations, high water‐retention capacity, and emulsifying capabilities, making it a promising alternative for food, petroleum, and cosmetic applications^17^. Recent studies have explored its use as a pH‐responsive delivery carrier for water‐ or fat‐soluble substances^17^, ^18^, a food additive^19^, and a novel natural gel modifier^20^, underscoring its significant potential. Notably, the sanxan‐producing strain natively co‐utilizes glucose and xylose without CCR.

With the development of synthetic biology, various microbial chassis cells have been used to produce high‐value natural products. Model strains such as *Escherichia coli* and *Saccharomyces cerevisiae* are regarded as advantageous chassis due to their well‐characterized genetic backgrounds and rich genetic toolkits^21^. Strain NXdP was hypothesized to be a potential and robust platform for the efficient, high‐value conversion of lignocellulose‐derived sugars through metabolic engineering and synthetic biology strategies. This study investigates the co‐utilization mechanism and metabolic pathways of glucose and xylose in strain NXdP. Subsequently, the glucose and xylose metabolic pathways in strain NXdP were reprogrammed via a “flux‐redirection” strategy. The growth, sanxan titer, productivity, and yield were assessed in a glucose–xylose mixture to compare the lignocellulose conversion potential of the original and final engineered strains. This work not only provides insights into the efficient conversion of lignocellulose but also offers key modular components (e.g., sugar transporters and xylose metabolic pathways) for the green production of other high‐value products. Furthermore, elucidation of the co‐utilization mechanism in the non‐model strain NXdP may inspire strategies to alleviate CCR in other bacteria and contribute to a broader understanding of microbial carbon metabolism.

## RESULTS

### Non‐PTS transporters are essential for both glucose and xylose uptake

To further explore the native co‐utilization mechanism and metabolic pathways of glucose and xylose in strain NXdP, a sanxan‐deficient strain (NXdPS) was constructed by knocking out four genes encoding glycosyltransferases (SsH) and polymerization and export proteins (SsG, SsC, and SsD) (Figure S1A). Phenotypic analysis on an agar plate (Figure S1B) and sanxan fermentation experiments (Figure S1C) confirmed successful disruption of sanxan biosynthesis in NXdPS.

CCR is a regulatory mechanism observed in most free‐living heterotrophic bacteria, primarily mediated by the PTS. In Gram‐negative bacteria such as *E*. *coli*, when glucose or other PTS substrates are present, the phosphoryl group from phosphoenolpyruvate (PEP) is transferred to sugars via PTS enzymes I (EI), histidine‐containing protein (HPr), and enzyme II domains A (EIIA) and BC (EIIBC)^22^. However, in strain NXdP, only the HPr component (encoded by gene *orf2260*) of the PTS was found^23^. Therefore, alternative transporters likely mediate the uptake of glucose and other sugars to compensate for the missing EIIBC components. Zhu et al. identified new xylose transporters that are not inhibited by glucose, enabling simultaneous consumption of both sugars^24^, highlighting the critical role of sugar transporters in alleviating CCR. Inspired by these findings, the mechanism of glucose and xylose co‐utilization in strain NXdPS was investigated from the perspective of sugar transporters.

Based on genome and comparative transcriptome analyses, over 70 genes encoding transporters were identified (Table S1). To focus on candidates likely involved in sugar uptake, only genes encoding Major Facilitator Superfamily (MFS) proteins or those annotated as sugar/general transporters were retained, yielding over 40 genes. By comparing transcript levels under glucose (Glc), xylose (Xyl), and mixed‐sugar (Glc–Xyl) conditions, genes with detectable expression (FPKM ≥ 5 in at least one condition) or significant expression differences were selected for further analysis. A final set of 13 genes (Table S2) was selected to investigate their roles in glucose and xylose uptake. Knockout strains for these genes were generated based on NXdPS. Using the wild‐type strain as a control, all strains were cultured in media containing glucose or xylose as the sole carbon source. Subsequently, their growth and carbon source utilization were measured (Figure S2). For most genes, knockout did not significantly affect sugar utilization. Notably, deletion of three specific genes (*orf0233*, *orf3653*, and *orf5235*) significantly altered growth rate and carbon source uptake efficiency. Among these, transporter 0233 had the most pronounced effect, as the growth (Figure 1A,G) and sugar utilization (Figure 1D,J) of the *orf0233* knockout strain (0*233* KO) showed a nearly stagnant state within 48 h. The *orf3653* knockout strain (*3653* KO) showed delayed growth (Figure 1B) and reduced glucose utilization (Figure 1E) only under glucose, while the *orf5235* knockout strain (*5235* KO) showed decreased growth (Figure 1C,I) and sugar utilization (Figure 1F,L) under both glucose and xylose. Growth and sugar utilization of all complemented strains recovered to levels comparable to those of the original strain. In addition, molecular docking verified the binding ability of these transporters to glucose and xylose (Figure S3). The binding energies were all below –1.2 kcal/mol, indicating feasible simulated binding. These results indicated that transporter 0233 may be the key transporter of glucose and xylose in the strain, while other transporters were also required for the uptake of both sugars.

**Figure 1:**
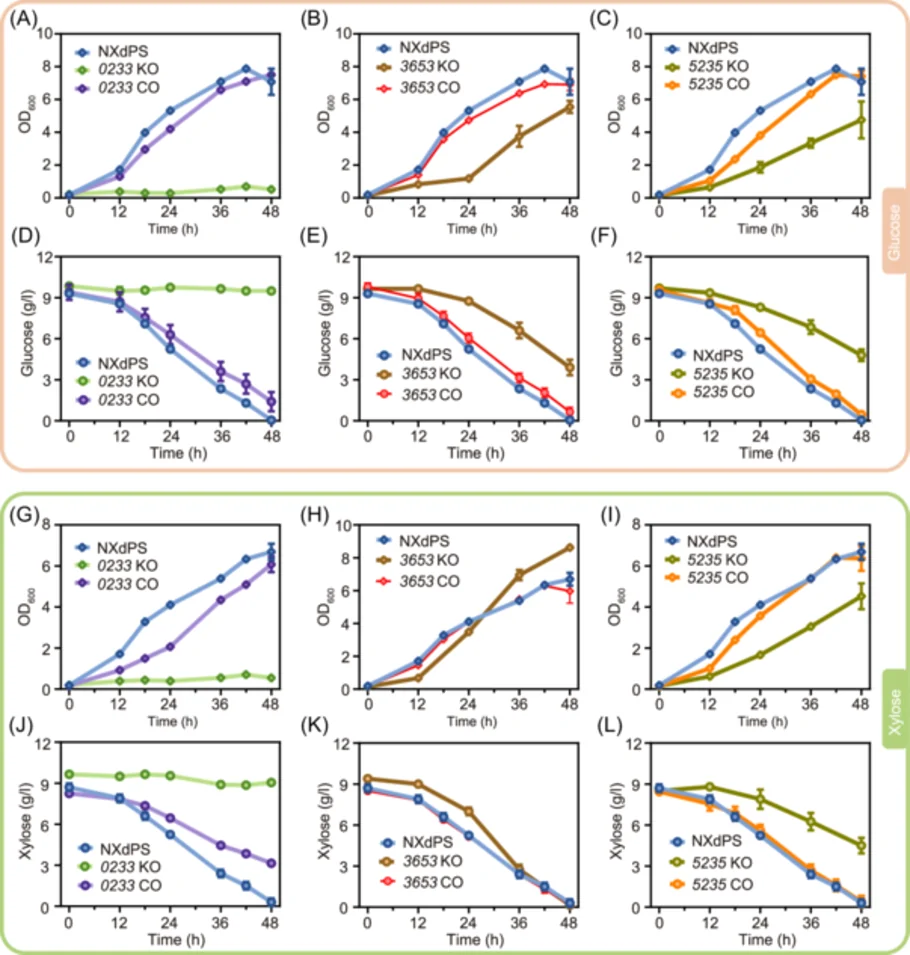
Non‐PTS transporters are essential for both glucose and xylose uptake. (A–C) Cellular growth time course of *Sphingomonas sanxanigenens* strains cultured in the seed medium containing 10 g/l glucose as the sole carbon source at 30°C and 250 rpm. (D–F) Corresponding glucose consumption of these strains in (A–C). NXdPS (control), *0233* KO, and *0233* CO strains were used in (A, D). NXdPS, *3653* KO, and *3653* CO strains were used in (B, E). NXdPS, *5235* KO, and *5235* CO strains were used in (C, F). (G–I) Cellular growth time course of *S*. *sanxanigenens* strains cultured in the seed medium containing 10 g/l xylose as the sole carbon source at 30°C and 250 rpm. (J–L) Corresponding xylose consumption of these strains in (G–I). NXdPS, *0233* KO, and *0233* CO strains were used in (G, J). NXdPS, *3653* KO, and *3653* CO strains were used in (H, K). NXdPS, *5235* KO, and *5235* CO strains were used in (I, L). Data represent mean ± SD (*n* = 3 biological replicates). CO,complemented; KO, knockout; PTS: phosphotransferase system.

### Entner–Doudoroff (ED) and Embden–Meyerhof–Parnas (EMP) pathways coexist in *S*. *sanxanigenens*

In the genome of NXdP, three key glucose metabolic pathways were found: (1) the periplasmic glucose oxidation (PGO) pathway, (2) the EMP pathway, and (3) the ED pathway (Figure 2A). The functions of key genes in each pathway were analyzed and verified. As shown in Figure 2A, glucose was taken up into the cell and partially catalyzed by glucokinase (GK) to form glucose‐6‐phosphate (G6P). Consequently, the GK‐encoding gene *orf0598* was knocked out in strain NXdPS, generating strain Δ*0598*. The growth curves and glucose utilization curves (Figure 2B) were measured with glucose as the carbon source. Strain Δ*0598* lost its ability to utilize glucose and grow, indicating that GK acts as a “master valve” in glucose metabolism in strain NXdP. If this valve is turned off, glucose metabolism cannot initiate. Furthermore, the gene *orf4389* encoding gluconokinase, a key enzyme in the PGO pathway, was knocked out in strain NXdPS. As shown in Figure 2C, the growth curves and glucose utilization curves of strain Δ*4389* were similar to those of the control strain NXdPS, suggesting that the PGO pathway was likely inactive in NXdPS.

**Figure 2:**
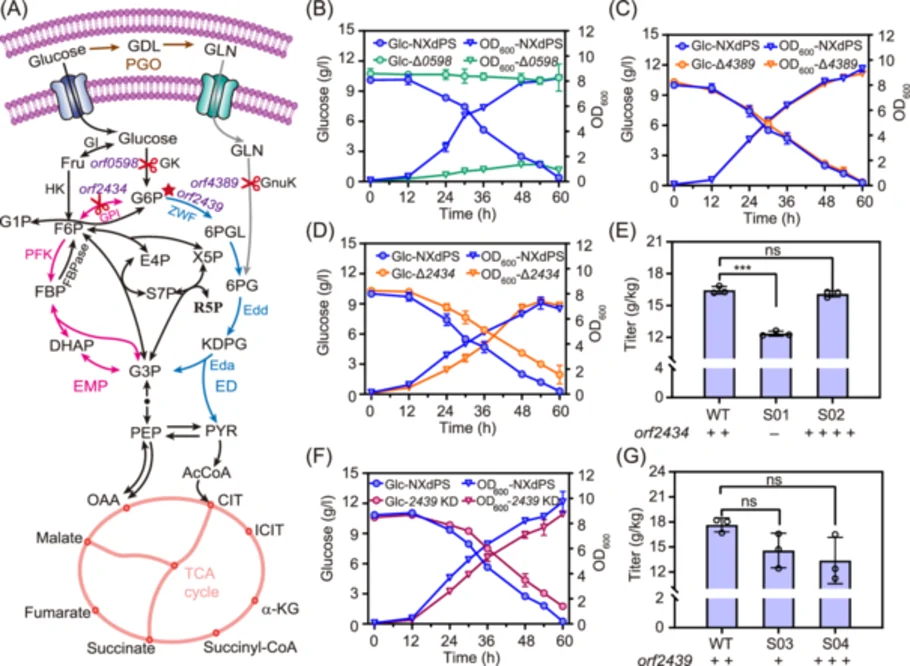
Coexistence of ED and EMP pathways in *S. sanxanigenens*. (A) Schematic diagram of the main glucose metabolic pathways (EMP, ED, and PPP) in *S*. *sanxanigenens* NXdP. The diagram was constructed based on whole‐genome annotation and transcriptome analysis from this study. (B–D) Cellular growth time course and glucose utilization curve of strains cultured in the seed medium containing 10 g/l glucose as the sole carbon source at 30°C and 250 rpm. Δ*0598* (knockout of *orf0598*, encoding glucokinase) (B), Δ*4389* (knockout of *orf4389*, encoding gluconokinase) (C), and Δ*2434* (knockout of *orf2434*, encoding glucose‐6‐phosphate isomerase) (D) were used. NXdPS was used as the control. (E) Sanxan titer of engineered strains with different expression levels of *orf2434*. WT: NXdP; S01: NXdP with *orf2434* knockout; and S02: NXdP with *orf2434* overexpression by plasmid. Cultures were grown in fermentation medium (40 g/l glucose) at 30°C and 250 rpm. (F) Cellular growth time course and glucose utilization curve of strains cultured in the seed medium containing 10 g/l glucose as the sole carbon source at 30°C and 250 rpm. *2439* KD (knockdown of *orf2439*, encoding glucose‐6‐phosphate dehydrogenase) were used. NXdPS was used as the control. (G) Sanxan titer of engineered strains with different expression levels of *orf2439*. S03: NXdP with *orf2439* knockdown; S04: NXdP with *orf2439* overexpression by genomic integration. Culture conditions are the same as in (E). Data represent mean ± SD (*n* = 3 biological replicates). “++” represents the native expression level of the gene in the wild‐type strain; “+” represents the expression level of the gene after its knockdown; “+++” represents the expression level of the gene after its overexpression via the knockin in the genome; “++++” represents the expression level of the gene after its overexpression via an expression vector; and “–” represents the expression level of the gene after its knockout. AcCoA, acetyl‐CoA; CIT, citrate; DHAP, dihydroxyacetone phosphate; E4P, erythrose‐4‐phosphate; ED: Entner–Doudoroff; EMP; Embden–Meyerhof–Parnas; Fru, d‐fructose; F6P, d‐fructose‐6‐phosphate; FBP, d‐fructose‐1,6‐bisphosphate; GDL, d‐glucono‐1,5‐lactone; G3P, d‐glyceraldehyde‐3‐phosphate; GLN, d‐gluconate; G1P, d‐glucose‐1‐phosphate; G6P, d‐glucose‐6‐phosphate; ICIT, isocitrate; KDPG, 2‐dehydro‐3‐deoxy‐d‐gluconate‐6‐phosphate; α‐KG, α‐ketoglutarate; OAA, oxaloacetate; PEP, phosphoenolpyruvate; PGO, periplasmic glucose oxidation; PPP, pentose phosphate; PYR, pyruvate; 6PGL, d‐glucono‐1,5‐lactone‐6‐phosphate; 6PG, d‐gluconate‐6‐phosphate; S7P, sedoheptulose‐7‐phosphate; TCA tricarboxylic acid.

Subsequently, the EMP and ED pathways were investigated via gene knockout experiments based on strain NXdPS. In the EMP pathway, glucose‐6‐phosphate isomerase (GPI), encoded by *orf2434*, plays a crucial role (Figure 2A). The defective strain Δ*2434* showed an initial growth lag but subsequently accelerated, reaching the stationary phase earlier than the control strain NXdPS, with no significant difference in the final biomass (Figure 2D). Additionally, its glucose utilization rate decreased. Knockout and overexpression of *orf2434* in strain NXdP yielded strains S01 and S02, respectively. Compared with strain NXdP, the sanxan titers of S01 and S02 decreased by 24.84% and 2.17%, respectively (Figure 2E). The glucose utilization ability of strain S01 (consuming 5.6 g/l glucose at 48 h) was lower than that of the control (consuming 6.7 g/l glucose at 48 h), whereas S02 (consuming 7.35 g/l glucose at 48 h) showed enhanced utilization (Figure S4). These results indicated that enhancing the EMP pathway could improve glucose utilization but was not beneficial for sanxan production. Glucose‐6‐phosphate dehydrogenase (ZWF) catalyzes the initial reaction when G6P enters the ED pathway. Knockout of *orf2439*, the gene encoding ZWF, was lethal, suggesting that the strain could not survive on the EMP pathway alone. A knockdown strain (*2439* KD) was constructed by integrating a low‐expressing copy of *orf2439* into the genome while deleting the native copy (Figure S5A). The relative transcript level of *orf2439* was downregulated in strain *2439* KD (Figure S5B), which also showed decreased growth and glucose utilization (Figure 2F). Knockdown and overexpression of *orf2439* in strain NXdP yielded strains S03 and S04, respectively. Compared with the control, the sanxan titers of S03 and S04 decreased by 17.40% and 24.23%, respectively (Figure 2G). An attempt to knock out *orf0597* (encoding phosphogluconate dehydratase, Edd) in the Δ*2434* background proved lethal. These results indicated that the ED pathway was active in strain NXdP and that directly interfering with it might cause metabolic imbalance, thereby affecting sanxan production.

### The ED pathway shows higher flux between parallel glycolytic pathways

Since metabolite levels and fluxes provide complementary information, analysis of both aspects yields a deeper understanding of metabolism^25^. Thus, [1‐^13^C] glucose was used as a tracer to cultivate strain NXdP to reveal the flux distribution between the EMP and ED pathways. The labeling patterns in key metabolites, which are used to decipher the flux distribution of metabolic networks, were recorded in the mass isotopomer distribution (MID) values of the derivatized fragments of proteinogenic amino acids^26^. The gas chromatography‐mass spectrum (GC‐MS) of serine (Ser) is shown in Figure S6A, ^27^. Furthermore, the theoretical metabolism with [1‐^13^C]‐glucose was analyzed and is illustrated in Figure S6B. [1‐^13^C]‐glucose generates metabolites with distinct labeling patterns via the EMP and ED pathways. For instance, the EMP pathway generates unlabeled (M + 0) and singly labeled (M + 1) glyceraldehyde‐3‐phosphate (G3P) in a 1:1 ratio, while the ED pathway generates only unlabeled (M + 0) G3P. Moreover, the position of isotope labeling in the resulting pyruvate differs between the two pathways. Theoretically, if the flux of the EMP pathway is 100%, the resulting Ser will be half unlabeled (M + 0) and half singly labeled (M + 1). Otherwise, if the flux of the ED pathway is 100%, Ser will be entirely unlabeled (M + 0)^28^. The experimentally measured proportion of unlabeled (M + 0) Ser was 97.07% ± 1.73% (Figure S6B), indicating a relatively stronger flux through the ED pathway than through the EMP pathway.

### Co‐existence of three xylose metabolic pathways enables efficient xylose metabolism

Our previous study revealed that strain NX02 contains three xylose metabolic pathways: (1) the xylose isomerase (XI) pathway, (2) the Weimberg pathway, and (3) the Dahms pathway^18^. The XI pathway is characterized by xylose isomerase (XylA) and xylulokinase (XylB), encoded by *orf1286* and *orf1287*, respectively. The resulting intermediate, xylulose, is phosphorylated to xylulose‐5‐phosphate (X5P) and enters the pentose phosphate (PPP) pathway. The Weimberg and Dahms pathways are both oxidative and share the same reactions up to 2‐dehydro‐3‐deoxy‐xylonate. First, periplasmic xylose is converted into xylonate by xylose dehydrogenase (XDH) and xylono‐1,5‐lactonase (XylC), which are encoded by *orf3261* and *orf3260*, respectively. Then, the intermediate 2‐keto‐3‐deoxy‐xylonate is formed by xylonate dehydratase (XD), encoded by *orf0234* or *orf4823*. In the Weimberg pathway, 2‐keto‐3‐deoxy‐xylonate dehydratase (KdxD, encoded by *orf3262*) converts it into 2,5‐dioxopentanoate; in the Dahms pathway, it is cleaved by an aldolase (AldL, encoded by *orf3841*) into pyruvate and glycolaldehyde (Figure 3A).

**Figure 3:**
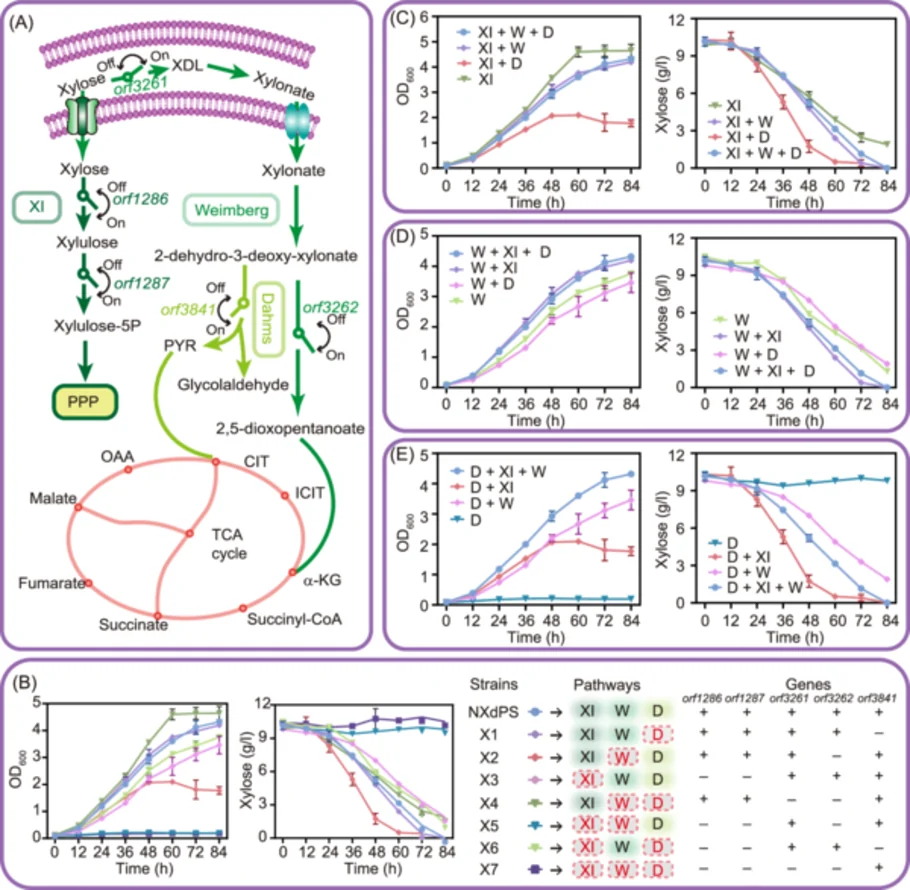
Three xylose pathways (XI, Weimberg, and Dahms) coexist in *S. sanxanigenens*. (A) Schematic diagram of the three xylose metabolic pathways, with key genes represented as “on–off” valves. The diagram was constructed from genome annotation and transcriptome data. (B–E) Cellular growth time course and xylose utilization curve of strains cultured in M9 medium containing 10 g/l xylose as the sole carbon source at 30°C and 250 rpm. (B) Overview of eight engineered strains (strains, genotypes, and the corresponding xylose metabolic pathways represented by each symbol shown in the inset legend). (C) Strains containing the XI pathway. (D) Strains containing the Weimberg pathway. (E) Strains containing the Dahms pathway. Data represent mean ± SD (*n* = 3 biological replicates). D, Dahms; W, Weimberg; XDL, d‐xylonolactone; XI, xylose isomerase.

Based on the xylose metabolism analysis, five key genes (*orf1286*, *orf1287*, *orf3261*, *orf3262*, and *orf3841*) were selected for knockout in strain NXdPS to construct the “on–off valves” for each pathway. Through individual or combined knockouts, eight strains with one, two, or no xylose pathways were constructed (named X1–X7; detailed genotypes are presented in Table S4). These strains were cultured with xylose as the sole carbon source, and growth and xylose utilization were measured at regular intervals. The growth curves, xylose utilization curves, genotypes, and corresponding pathways (symbols in the figure) are shown in Figure 3B. Notably, the strain lacking all xylose pathways and the strain retaining only the Dahms pathway both failed to grow and utilize xylose. To clearly compare the XI, Weimberg, and Dahms pathways, the strain possessing all three pathways served as a control, and strains sharing the same pathway were grouped together. Interestingly, the strain with only the XI pathway reached the stationary phase earlier (60 h, OD_600_: 4.60) and consumed 6.0 g/l xylose, showing increased growth with less xylose consumption than the control (OD_600_: 3.60; xylose consumption: 7.0 g/l). The strain with the XI and Weimberg pathways showed growth (OD_600_: 3.76 at 60 h) and xylose consumption (7.9 g/l) similar to those of the control. However, the growth (OD_600_: 2.09 at 60 h) of the strain with the XI and Dahms pathways decreased, while the xylose consumption (9.8 g/l) increased significantly (Figure 3C). These results indicated that the XI pathway was highly efficient when acting alone, whereas the presence of the Dahms pathway slowed overall growth. Similar observations were made for the combination of the Weimberg and Dahms pathways (Figure 3D). In any combination of two xylose pathways, the coexistence of the Dahms pathway reduced the overall growth rate (Figure 3B,E). Furthermore, the Dahms pathway alone was insufficient to support bacterial growth on xylose. Based on these results, it could be inferred that among the three xylose pathways, the XI pathway is the most potent, followed by the Weimberg pathway, while the Dahms pathway is the weakest. The coordinated action of these three xylose pathways with varying strengths enables efficient xylose metabolism in strain NXdPS.

### Glucose and xylose co‐transporters and efficient metabolic network enable co‐utilization

The reliance on non‐PTS transporters for carbon uptake is a foundational prerequisite for synergistic glucose and xylose utilization in strain NXdP, while its glucose and xylose metabolic networks constitute another critical aspect of the co‐utilization mechanism. Based on the above results, the mechanism of glucose and xylose co‐utilization in strain NXdPS can be summarized into three points: First, the PTS for glucose uptake in this strain is incomplete, meaning that the presence of glucose cannot inhibit the uptake and utilization of other carbon sources; second, there are multiple non‐PTS transporters involved in the uptake of both glucose and xylose, which ensures the efficient uptake of sugars; and lastly, the strain possesses the EMP and ED pathways for glucose metabolism and three xylose metabolic pathways with varying strengths. Therefore, the ability to utilize both carbon sources is robust enough to result in the phenomenon of synergistic utilization. Furthermore, the schematic pathways of glucose and xylose metabolism, along with the sanxan biosynthesis pathway in strain NXdP, are depicted in Figure 4.

**Figure 4:**
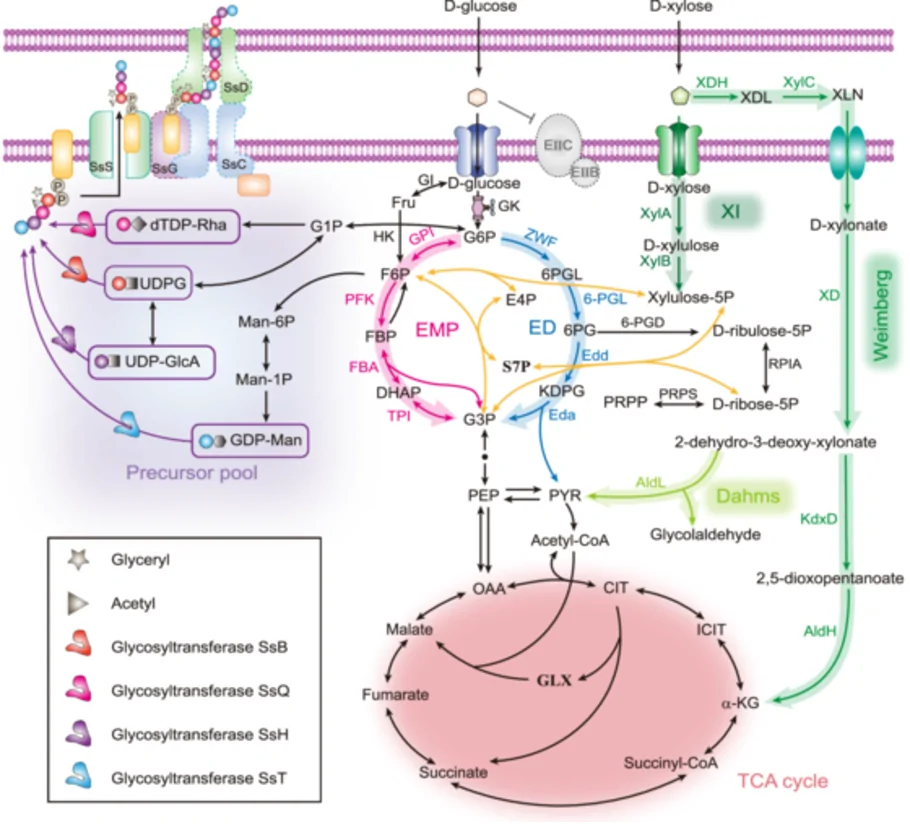
Glucose and xylose co‐transporters and an efficient metabolic network enable co‐utilization of glucose and xylose in *S. sanxanigenens*. Schematic diagram of the glucose and xylose network for sanxan production is shown. This diagram was constructed based on the whole‐genome annotation and transcriptome analysis. Arrows indicate the pathway steps existed in strain NXdP. Pink arrows indicate the unique pathways of EMP before it converges with the ED pathway. Blue arrows indicate the unique pathways of ED before it converges with the EMP pathway. Green arrows (from dark to light in color) indicate the pathways of XI, Weimberg, and Dahms in sequence. Yellow arrows indicate the rest of the pathways of pentose phosphate (PPP) other than those shared with the ED pathway. Dotted gray lines represent the missing key PTS components. dTDP‐L‐Rha, deoxythymidine diphosphate‐L‐rhamnose; GLX, glyoxylate; GDP‐Man, guanosine diphosphate‐mannose; Man‐6P, d‐mannose‐6‐phosphate; Man‐1P, D‐mannose‐1‐phosphate; UDPG, uridine diphosphate glucose; UDP‐GlcA, uridine diphosphate glucuronate; and XLN, d‐xylonate.

### The sanxan biosynthesis is improved via a “flux‐redirection” strategy

Lignocellulosic biomass is the most abundant renewable carbon resource on Earth. The two main sugars in its hydrolysate are glucose and xylose, which can serve as carbon sources for microbial fermentation^29^. A previous study reported that xylose reduces sanxan production, with higher xylose content leading to lower titers^23^. Therefore, based on the glucose and xylose metabolic pathways (Figure 4), the metabolism of strain NXdP was designed and optimized to construct an engineered strain with improved sanxan titer and enhanced glucose and xylose utilization.

First, the glucose metabolic pathways in strain NXdP were optimized. As shown in Figure 5A, the EMP pathway, the ED pathway, and the tricarboxylic acid (TCA) cycle are the main glucose routes. The above results demonstrated that attenuating or enhancing either the EMP or ED pathway reduces sanxan synthesis. Since reducing carbon flux from G6P to branch pathways other than polysaccharide synthesis failed to increase polysaccharide production, increasing the G6P pool is another strategy to enhance sanxan production. GK functions as the main valve controlling cellular glucose flux into G6P. The gene *orf0598* encoding GK was overexpressed in strain NXdP, resulting in strain S05. Unexpectedly, sanxan production by S05 decreased by 49.86% compared with NXdP, nearly half (Figure 5B). This may be due to G6P accumulation resulting from GK overexpression, which exerts feedback inhibition on upstream enzymes such as hexokinase, thereby hindering further glucose phosphorylation and utilization. A supplementary glycolytic pathway from glucose to the EMP pathway via fructose was identified in strain NXdP. This pathway diverts the glucose immediately upon entry, and therefore, an attempt was made to block it. This pathway consists of two reactions: (1) Glucose is catalyzed into fructose by glucose isomerase (GI), and its encoding genes, *orf0650* and *orf0655*, are part of the same operon. (2) Fructose is catalyzed into fructose‐6‐phosphate (F6P) by hexokinase (HK, encoded by the gene *orf1342*). The entire GI operon (*orf0649*‐*orf0656*) and the gene *orf1342* were individually knocked out in strain NXdP, resulting in strains S06 and S07, respectively. Sanxan production by strains S06 and S07 increased by 9.62% and 3.97%, respectively (Figure 5C,D). Thereafter, combined knockout of *orf0649*–*orf0656* and *orf1342* increased sanxan titer by 9.95% in strain S08 (Figure 5E). Given that polysaccharide synthesis is linked to substrate uptake and metabolism, the transporters identified above (0233, 3653, and 5235) were overexpressed in strain S08. However, sanxan titer decreased non‐significantly (Figure S7). Thus, the strain S08 was selected for subsequent experiments.

**Figure 5:**
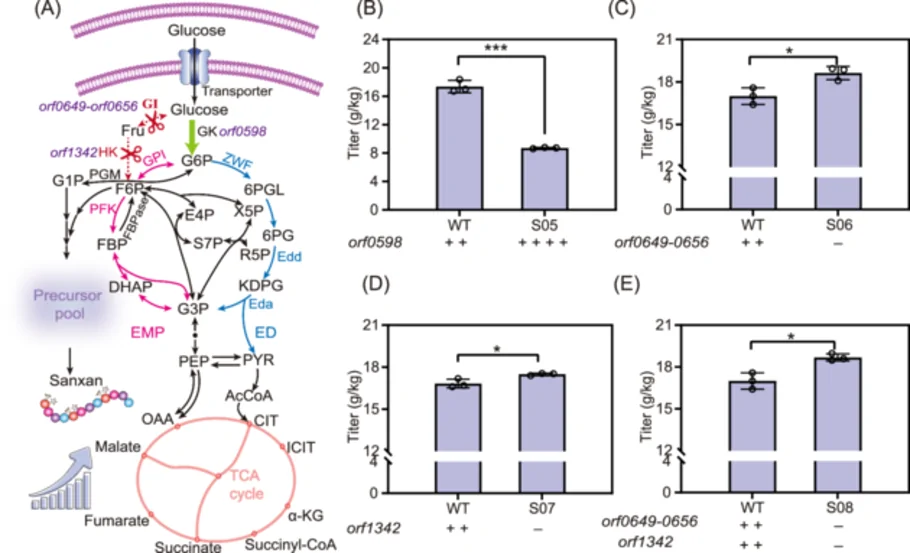
Blocking the supplementary glycolytic pathway from glucose to fructose‐6‐phosphate increases sanxan titer. (A) Schematic of the metabolic network for sanxan biosynthesis from glucose. Dotted red arrows indicate the pathways abolished by the deletion of GI and HK. The green arrow indicates the pathway strengthened by overexpression of GK. The diagram was constructed based on the whole‐genome annotation and transcriptome analysis. (B–E) Sanxan titer of engineered strains cultured in the fermentation medium containing 40 g/l glucose as the sole carbon source at 30°C and 250 rpm. (B) Strains with different expression levels of *orf0598* (encoding GK). WT: NXdP; S05: NXdP with *orf0598* overexpression by plasmid. (C) Strains with different expression levels of *orf0649*–*0656* (encoding GI operon). S06: NXdP with *orf0649*–*orf0656* knockout. (D) Strains with different expression levels of *orf1342* (encoding HK). S07: NXdP with *orf1342* knockout. (E) Strains with different expression levels of *orf0649*–*orf0656* and *orf1342*. S08: NXdP with *orf0649*–*orf0656* and *orf1342* knockout. All data represent mean ± SD (*n* = 3 biological replicates). Statistical analysis was performed using Student's *t*‐test with strain NXdP as the control. *p < 0.05; ***p < 0.001. “++” represents the native expression level of the gene in the wild‐type strain; “++++” represents the expression level of the gene after its overexpression via an expression vector; and “–” represents the expression level of the gene after its knockout.

To engineer a strain capable of efficiently co‐utilizing glucose and xylose for sanxan production, strains possessing distinct xylose metabolic pathways were developed based on S08. As presented in Figure 4, the Dahms pathway produces glycolaldehyde, which is not recycled into central carbon metabolism, leading to carbon loss. Among the three xylose pathways, the Dahms pathway shows the lowest activity and slows cell growth. Therefore, it was eliminated in strain S08, and the Weimberg pathway was retained because it enters the TCA cycle with fewer steps than the XI pathway, resulting in strain S09. According to the xylose pathway analysis, the strain retaining only the XI pathway showed the highest growth rate. Consequently, strain S10, which retained solely the most efficient XI pathway, was constructed. A simplified diagram illustrating glucose and xylose metabolism in strains S08, S09, and S10 is presented in Figure 6A. Subsequently, strains S08, S09, and S10 were fermented under three carbon source conditions: (1) glucose; (2) xylose; and (3) a mixture of glucose and xylose. Herein, the mixed sugar contained approximately 33 g/l glucose and 7 g/l xylose, mimicking the ratio in lignocellulosic hydrolysate. As shown in Table 1 and Figure 6B, strain S09 produced 18.64 ± 0.30 g/kg sanxan on glucose, significantly higher than the control (17.11 ± 0.50 g/kg, *p* < 0.05). This suggested that elimination of the Dahms pathway might enable more efficient sanxan synthesis. Conversely, on xylose alone, strain S10 achieved the highest titer (10.31 ± 0.45 g/kg), an 8.69% increase over the control (Figure 6C). This may be because the highly efficient XI pathway can supply more carbon flux for sanxan synthesis than needed for growth. Under mixed sugars, strain S09 produced 18.16 ± 0.46 g/kg sanxan, 8.94% higher than the control (16.67 ± 0.23 g/kg, *p* < 0.01). Moreover, strain S09 consumed amounts of glucose and xylose similar to those of the control strain (Figure 6D). Overall, strain S09, developed via a “flux‐redirection” strategy, performed best on mixed sugars and was selected as the final engineered strain for further study.

**Figure 6:**
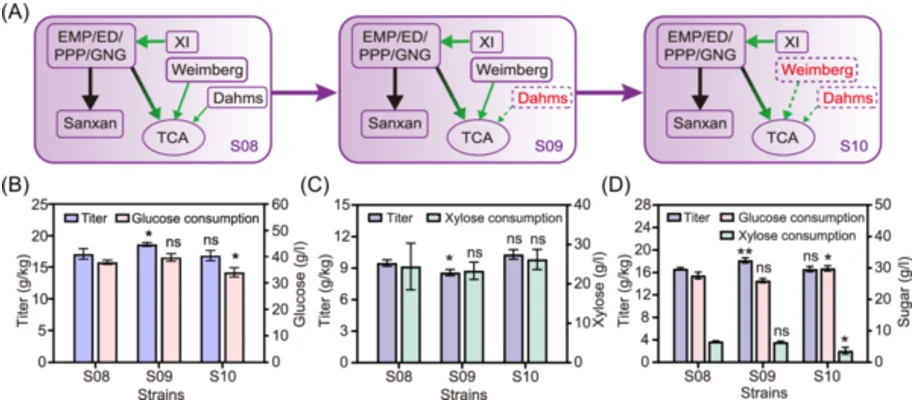
Integration of modifications in glucose and xylose metabolic pathways improves sanxan production. (A) A brief diagram of glucose and xylose metabolism existed in strains S08, S09, and S10. This schematic was generated based on the analysis of the metabolic network of *S. sanxanigenens* NXdP (this study). S08: NXdP with *orf0649*–*orf0656* (encoding glucose isomerase operon) and *orf1342* (encoding hexokinase) knockout (possessing XI, Weimberg, and Dahms pathways); S09: S08 with *orf3841* (encoding aldolase) knockout (possessing XI and Weimberg pathways); S10: S08 with *orf3261*–*orf3262* (encoding xylose dehydrogenase and 2‐keto‐3‐deoxy‐xylonate dehydratase, respectively) knockout (possessing only the XI pathway). (B–D) Sanxan titer and consumed sugars of engineered strains in shake flask fermentation in fermentation medium at 30°C and 250 rpm. (B) Sole glucose (40 g/l). (C) Sole xylose (40 g/l). (D) Mixed glucose (33 g/l) and xylose (7 g/l). Data are mean ± SD (*n* = 3 biological replicates). Statistical analysis was performed using Student's t‐test, with strain S08 as the control, under the same substrate conditions. *p < 0.05; **p < 0.01.

**Table 1:** Comparison of sanxan titer and consumed sugars of engineered strains in shake flask fermentation.

Strain	Substrates	ΔGlc (g/l)	ΔXyl (g/l)	Sanxan titer (g/kg)
S08	Glc	37.60 ± 0.02	–	17.11 ± 0.50
	Xyl	–	24.40 ± 5.92	9.48 ± 0.31
	Glc and Xyl	27.60 ± 1.06	6.63 ± 0.22	16.67 ± 0.23
S09	Glc	39.73 ± 1.51	–	18.64 ± 0.30*
	Xyl	–	23.33 ± 2.20	8.58 ± 0.29*
	Glc and Xyl	26.00 ± 0.69	6.41 ± 0.35	18.16 ± 0.46**
S10	Glc	34.00 ± 1.74	–	16.84 ± 0.85
	Xyl	–	26.60 ± 2.55	10.31 ± 0.45
	Glc and Xyl	29.87 ± 0.83*	3.68 ± 1.09*	16.60 ± 0.47

ΔGlc (%) = (total glucose consumed/initial total glucose) × 100%; ΔXyl (%) = (total xylose consumed/initial total xylose) × 100%. Statistical analysis was performed using Student's *t*‐test, with strain S08 as the control, under the same substrate conditions. *p < 0.05; **p < 0.01.

### The engineered strain S09 shows excellent performance in scale‐up fermentation with mixed sugars

To evaluate the fermentation performance of engineered strain S09 at a larger scale, batch fermentations were conducted in 5 l bioreactors using a defined sugar mixture (33 g/l glucose and 7 g/l xylose) that mimics the major sugar composition of lignocellulosic hydrolysate, with the original strain NXdP as the control. The fermentation kinetic curves of strains NXdP and S09 are shown in Figure 7A,B, respectively. During the first 12 h, strain S09 showed significantly better growth than the control, with no apparent lag phase. After 12 h, the strain entered a stage where growth and sanxan synthesis proceeded simultaneously. Finally, the sanxan titer of strain NXdP reached 17.84 ± 0.37 g/kg, with a cell dry weight (CDW) of 2.15 ± 0.07 g/kg. The viscosity of the fermentation broth (Table S3) followed the same trend as the sanxan titer, and the viscosity of the S09 fermentation broth was 5.48% higher than that of NXdP at 84 h. The yield and productivity of sanxan for strain NXdP were 0.44 ± 0.01 g/g carbon source and 0.21 ± 0.00 g/kg/h, respectively. In comparison, the engineered strain S09 achieved a higher sanxan titer of 20.25 ± 0.78 g/kg, with a CDW of 2.70 ± 0.14 g/kg, and corresponding yield and productivity of 0.50 ± 0.02 g/g carbon source and 0.24 ± 0.01 g/kg/h, respectively. Additionally, the comparison of glucose and xylose utilization efficiency between strains NXdP and S09 during fermentation is shown in Table 2. Notably, strain S09 showed higher glucose utilization efficiency than strain NXdP before 48 h; after xylose depletion, the glucose utilization efficiency of S09 is comparable to that of NXdP. Strain S09 also showed enhanced xylose utilization efficiency (except during the initial 12 h), indicating that deletion of the Dahms pathway was an effective strategy to enhance xylose utilization. After reprogramming the glucose and xylose metabolic pathways, strain S09 not only grew faster on the mixture of glucose and xylose but also increased the sanxan titer by 13.51% ± 4.38%.

**Figure 7:**
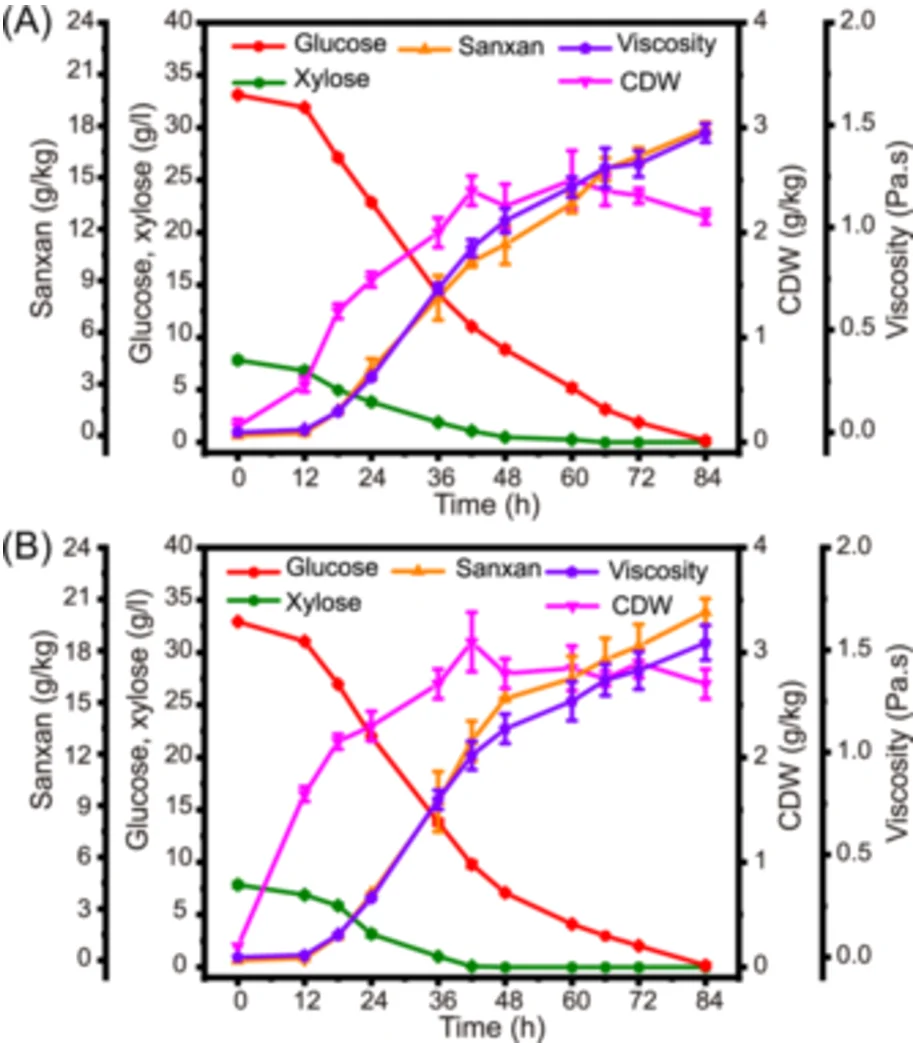
The engineered strain S09 shows enhanced sanxan production in scale‐up fermentation with mixed glucose and xylose. Fermentation kinetics curves of strains cultured in 5 l bioreactors with a sugar mixture containing 33 g/l glucose and 7 g/l xylose (total 40 g/l) are shown. Conditions: 30°C, pH 7.0, agitation 300–600 rpm, and aeration 1 vvm. (A) Strain NXdP (original). (B) Strain S09 (engineered). Data are mean ± SD (*n* = 3 biological replicates). CDW, cell dry weight.

**Table 2:** Comparison of glucose and xylose utilization efficiency of strains NXdP and S09 grown on a co‐substrate at different time intervals in 5 l fermenters.

Time	Strain	ΔGlc (%)	ΔXyl (%)	ΔSugar (%)
12 h	NXdP	3.62 ± 0.29	13.01 ± 0.48	5.42 ± 0.77
	S09	5.71 ± 0.18***	12.10 ± 0.35	6.94 ± 0.53
24 h	NXdP	30.94 ± 0.36	50.89 ± 0.44	34.76 ± 0.80
	S09	33.17 ± 0.11**	59.62 ± 0.45***	38.26 ± 0.56**
36 h	NXdP	57.21 ± 0.43	75.64 ± 0.36	60.73 ± 0.79
	S09	58.06 ± 0.32	86.88 ± 0.25***	63.60 ± 0.57**
48 h	NXdP	73.30 ± 0.39	93.75 ± 0.25	77.21 ± 0.64
	S09	78.45 ± 0.21***	100.00 ± 0.21***	82.60 ± 0.42***
72 h	NXdP	94.28 ± 0.32	100.00 ± 0.23	95.37 ± 0.55
	S09	93.78 ± 0.14	100.00 ± 0.21	94.98 ± 0.35

ΔGlc (%) = (total glucose consumed/initial total glucose) × 100%; ΔXyl = (total xylose consumed/initial total xylose) × 100%; and ΔSugar = (total sugar consumed/initial total sugar) × 100%. Statistical analysis was performed using Student's *t*‐test, with strain NXdP as the control, at the same time points and under the same substrate conditions. **p < 0.01; ***p < 0.001.

To further explore the mechanism underlying the enhanced production of strain S09 compared with NXdP, bacterial cells from both strains were collected at 24 h (mid‐exponential growth phase) and analyzed for intracellular metabolites related to CCM. Due to technical limitations, only a subset of CCM metabolites was detected. Based on the results, a schematic diagram (Figure 8) was constructed to illustrate changes in metabolite contents in strain S09 relative to the original strain NXdP. First, after reprogramming glucose and xylose metabolism, the contents of Glc, glucose‐1‐phosphate (G1P), and mannose‐6‐phosphate (Man‐6P) increased by 2.71 ± 1.00, 2.16 ± 0.22, and 2.53 ± 0.75 times, respectively, suggesting enhanced flux toward precursor synthesis. This may be an important factor contributing to the improved sanxan titer. Second, the decreased levels (approximately 0.81‐fold) of G6P, F6P, and fructose‐1,6‐bisphosphate (FBP) indicated downregulation of the flux from glucose to the EMP pathway, meaning that metabolic flux diversion from G6P to the EMP pathway was weakened. Third, a portion of xylose entered the PPP pathway via the XI pathway, while the remainder was routed into the TCA cycle through the Weimberg pathway. The increased contents of G3P, 3‐phosphoglyceric acid (3PG), and α‐ketoglutarate (α‐KG) suggested sufficient carbon flux from xylose, which may be associated with accelerated strain growth.

**Figure 8:**
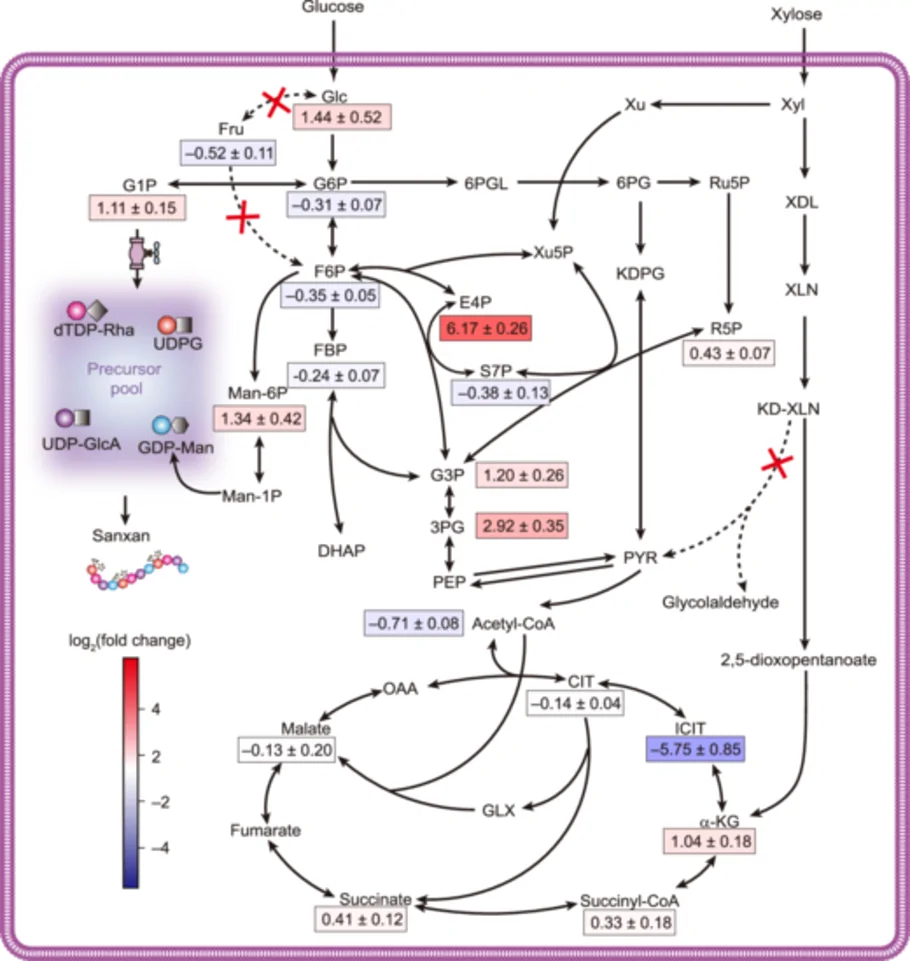
Enhanced precursor synthesis and carbon flux optimization via metabolic reprogramming are the intrinsic mechanisms for increased sanxan production in engineered strain S09. This schematic was generated based on the CCM analysis of the engineered strain S09 relative to the original strain NXdP during fermentation in the sugar mixture. Dotted black arrows and red cross indicate pathways that were deleted in S09. All values in the figure represent the log_2_(fold change) of the metabolite content in strain S09 relative to the control strain NXdP, corresponding to the base‐2 logarithm of the fold change. CCM, central carbon metabolism; Glc, glucose; KD‐XLN, 2‐dehydro‐3‐deoxy‐xylonate; Xyl, xylose; Xu, xylulose; Xu5P, xylulose‐5‐phosphate.

Overall, strain S09 has compensated for the deficiency of the original strain NXdP, which showed decreased production when cultured with xylose. More carbon flux was successfully directed to sanxan biosynthesis and cell growth in the engineered strain S09, with minimized carbon loss. Moreover, it has demonstrated high production levels in mixed sugar fermentation, making it attractive for industrial applications and the biological conversion of lignocellulose.

## DISCUSSION

In this study, we demonstrated that strain NXdP harbors diverse sugar transporters that play a key role in the co‐utilization of glucose and xylose. This discovery provides new insights into the functional mechanisms of *Sphingomonas* and may be extended to other bacterial systems, pending heterologous validation. Moreover, strain NXdP possesses two main glucose pathways and three xylose pathways with distinct metabolic activities, enabling efficient sugar utilization and providing useful modules for the green synthesis of other high‐value products. Most conclusions were drawn from studies using the non‐sanxan‐producing strain NXdPS, where sanxan removal may alter carbon partitioning, energy balance, and global regulation. However, this modification does not affect the fundamental machinery for glucose and xylose uptake. Therefore, the sugar transporters identified in NXdPS are also applicable to its parental strain, NXdP. As shown in the metabolic network (Figure 4), after entering the cell, glucose is partitioned at the G6P node: one portion flows toward sanxan synthesis via G1P, while the remaining carbon flux is allocated to the EMP and ED pathways. Among the xylose pathways, only the XI pathway intersects with glucose metabolism via the PPP pathway and does not converge with sanxan synthesis. Therefore, the presence of the sanxan synthesis pathway likely does not affect the metabolic activities of the three xylose pathways. This inference is indirectly supported by fermentation results from strains engineered with modified xylose pathways in the NXdP background, which showed trends consistent with those in NXdPS. Furthermore, experiments investigating flux through the EMP and ED pathways were conducted in the original strain NXdP, and the conclusions accounted for the influence of sanxan synthesis on the overall metabolic network.

A previous study reported that xylose in the fermentation medium reduced sanxan titer^23^. This phenomenon has also been observed in other bacteria. For instance, Temudo et al. reported that during xylose fermentation by mixed microbial cultures, a larger carbon fraction was converted into catabolic products (butyrate, acetate, and ethanol), resulting in an approximately 20% lower biomass yield than from glucose^30^. Similarly, Shi et al. observed decreasing chondroitin titers in *Corynebacterium glutamicum* with increasing xylose ratios^31^. Analogous reductions have been reported for ethanol production. For example, *S*. *cerevisiae* 424A (LNH‐ST) produced lower ethanol titers on glucose–xylose mixtures than on glucose alone;^32^ *Pachysolen tannophilus* produced 5.5% ethanol from 12% glucose but only 2% from 12% xylose^33^. Collectively, these findings demonstrate that xylose‐associated titer reduction is a widespread phenomenon across prokaryotes and eukaryotes. To address this issue, an engineered strain S09 that showed better performance on mixed sugars (glucose and xylose, the major components of lignocellulosic hydrolysate) was constructed via a “flux‐redirection” strategy. The enhanced performance of strain S09 likely stems from two complementary mechanisms. First, deletion of the Dahms pathway eliminates a carbon dissipation route. Second, metabolic network optimization redirects metabolic flux toward sanxan synthesis.

Improvement of viscous polysaccharide production is hindered by low substrate conversion efficiency, high by‐product formation^34^, and insufficient dissolved oxygen and difficult substrate uptake caused by high viscosity. Mutagenesis breeding is a common approach to obtain superior mutant strains with the desired traits. For example, Sun et al. used Ultraviolet‑Atmospheric and Room‐Temperature Plasma (UV‐ARTP) combined mutagenesis to obtain a low‐molecular‐weight gellan‐producing mutant of *Sphingomonas paucimobilis* ATCC 31461, increasing gellan titer by 24%^35^. UV‐ARTP mutagenesis also yielded a welan‐producing mutant with an 83.54% higher titer than the original strain, although the final titer was only 17.40 g/l^36^. Synthetic biology offers another strategy to improve the performance of bacterial strains for viscous polysaccharide production. Manjusha et al. constructed recombinant *S*. *paucimobilis* ATCC 31461 strains overexpressing cloned gellan biosynthetic genes; the strain overexpressing *gelQ* produced the highest gellan titer (11.71 ± 0.2 g/l, an increase of 83.83%)^37^. It is worth noting that the strain NXdP used in this study had already undergone rational and random genetic manipulations to enhance sanxan production, achieving a 42.5% increase in a previous study^16^. Further improvements in an already optimized chassis are typically more difficult to achieve, a phenomenon known as the “diminishing returns” effect in metabolic engineering^38^. Moreover, unlike the gellan study, which started from a low baseline (6.37 ± 0.3 g/l) and achieved 11.71 ± 0.2 g/l (a large relative increase but a modest absolute gain of 5.34 g/l), this study achieved an absolute titer increase of 2.41 g/l (from 17.84 to 20.25 g/l) from a relatively high baseline. Given the well‐documented challenges of improving viscous polysaccharide titers, the relatively high baseline of the parent strain, and its prior engineering, the 13.51% ± 4.38% improvement achieved here represents a significant advance toward efficient sanxan production.

Overall, this study provides a highly promising platform strain for efficient co‐utilization of glucose and xylose and identifies key modular components (e.g., sugar transporters, precursor synthesis pathways, and xylose metabolic pathways) that may guide the engineering of other strains for green production of high‐value products.

## MATERIALS AND METHODS

### Bacterial strains and plasmids

The main strains and plasmids used in this study are listed in Table S4. *S. sanxanigenens* NXdP (CGMCC 15406) was used as the parental strain. *E*. *coli* S17 was used for culturing recombinant plasmids for gene knockout and overexpression. Genes were knocked in and knocked out by double‐crossover homologous recombination. High‐fidelity enzyme PrimeSTAR Max DNA Polymerase (TaKaRa) were used for DNA amplification. The upstream and downstream flanking sequences of the target gene, together with gene fragments (only for the genes that need to be knocked in), were ligated into vector pLO3^39^ using the Gibson Assembly method (NEB) following the standard protocol. The recombinant plasmids, sequenced by GENEWIZ Biotechnology Co., Ltd, were chemically transformed into *S*. *sanxanigenens*. Chromosomal gene knockouts were conducted using a *sacB*‐negative counter‐selection system, followed by verification using PCR. Similarly, the expression plasmids were constructed using vector pBT^40^ and transformed into *S*. *sanxanigenens* strains by biparental conjugational transfer, and then verified by PCR.

### Media and cell cultivation

NK medium (peptone 5.0 g/l, beef powder 3.0 g/l, and yeast extract 1.0 g/l, pH 7.0) supplemented with a related carbon source was used to preliminarily cultivate *S*. *sanxanigenens* NXdP and its derivative strains at 30°C and 250 rpm. For cell growth measurement, the seed medium (glucose 10 g/l, beef powder 5.0 g/l, peptone 3.0 g/l, and yeast extract 1.0 g/l, pH 7.0) was used, supplemented with related sugars (glucose or xylose). Specifically, M9 medium (Na_2_HPO_4_ 6.78 g/l, KH_2_PO_4_ 0.6 g/l, NaCl 0.1 g/l, and NH_4_Cl 0.2 g/l) supplemented with 2 mM CaCl_2_, 40 mM MgSO_4_, and 10 g/l xylose was used for the characterization of three xylose metabolic pathways. The fermentation medium (carbon source 40.0 g/l, yeast extract 0.2 g/l, K_2_HPO_4_ 1.2 g/l, NaNO_3_ 2.0 g/l, CaCO_3_ 1.0 g/l, FeSO_4_ 0.005 g/l, NaCl 0.4 g/l, and MgSO_4_ 0.5 g/l, pH 7.5) was used for sanxan production. Luria–Bertani (LB) medium (5 g/l yeast extract, 10 g/l peptone, and 10 g/l NaCl) was used to cultivate the *E*. *coli* S17 at 37°C and 200 rpm.

### RNA isolation and chain‐specific transcriptome sequence

To identify transporters that are related to the uptake of glucose and xylose, the changes in the whole‐transcriptome profile of strain NXdP cultured in different carbon sources, including Glc, Xyl, and a 1:1 Glc–Xyl mixed substrate, were investigated and compared. First, a starter culture was prepared by incubating the strain NXdP in NK medium at 30°C for approximately 12 h, followed by inoculation at 5% (v/v) into the seed medium and incubation at 30°C for a further 12 h. Next, the seed broth was inoculated at 10% (v/v) into the fermentation medium with the aforementioned carbon sources. The samples were harvested when cultivated at 36 h by centrifugation at 5000*g* for 5 min at 4°C. The crude total DNA‐free RNA of the cell pellets was extracted using the RNAiso Plus (TaKaRa) and RNAprep Pure Cell/Bacteria Kit (Tiangen). Quantification of gene transcript levels was performed at the Novogene Bioinformatics Technology Co., Ltd.

### ^13^C labeling and analysis

The process of cell culture and amino acid extraction, and derivatization was carried out according to the protocol described in the study of You, et al., with minor modification^41^. Briefly, strain NXdP was first grown in a nonlabeled M9 medium, and the middle‐log growth phase cells were inoculated at 3% (v/v) into the M9 medium with 3 g/l [1‐^13^C] glucose (purity = 99%, Macklin). The labeled culture was sub‐cultured at 3% (v/v) in the same labeled medium. The sub‐cultured cells in the middle‐log growth phase were harvested by centrifugation at 8000*g* for 10 min. The pellet was resuspended in 1.5 ml of 6 M HCl, transferred to a clear glass, screw‐top GC vial, and then incubated at 100°C for 24 h. After that, the samples were centrifugated at 14,000*g* for 10 min using 2 ml of Eppendorf tubes, and the supernatants were transferred to new GC vials. The liquid was freeze‐dried and then dissolved with 150 μl of tetrahydrofuran (Sigma‐Aldrich) and 150 μl of N‐(tert‐butyl dimethylsilyl)‐N‐methyl‐trifluoroacetamide (Macklin). Thereafter, all samples were incubated in an oven at 70°C for 1 h with occasional mixing. The prepared samples were then analyzed by Agilent 7890A‐5975C GC‐MS (use a split ratio = 1:10, carrier gas helium = 1.2 ml/min, and injection volume = 1 μl) equipped with an HP‐5MS GC column (Hewlett‐Packard). The GC temperature program was as follows: hold at 150°C for 2 min, increased at 3°C/min to 280°C and at 20°C/min to 300°C, and then hold for 5 min. Mass spectra were acquired over an *m/z* range of 60–500. Raw MID data of derivatized samples were recorded and subsequently processed using a software package to correct for natural isotope abundances^26^.

### Molecular docking

First, the PDB files of the simulated three‐dimensional (3D) models of the transporters 0233, 3653, and 5235 were obtained from the protein database Uniprot (https://www.uniprot.org). Subsequently, the PDB files of the 3D models of the ligand were downloaded from the PubChem database (https://pubchem.ncbi.nlm.nih.gov/). Autodock (version 4.2.6) was used for molecular docking and calculation of the binding affinity between each potential sugar transporter and ligand (glucose and xylose). By default, the software uses a semi‐flexible docking algorithm, which allows the ligands to sample various conformations during a semi‐flexible docking stage, whereas the protein receptor remains rigid^42^. The docking results between the small ligand molecules and the protein targets were observed using PyMOL software (Version 2.2.0, https://pymol.org/), and the polar contacts to other atoms in object were analyzed^43^.

### Shake flask cultivation for sanxan production

*S. sanxanigenens* was activated on NKG agar (NK medium with 15 g/l glucose and 15.0 g/l agar) and incubated at 30°C for about three days. A single colony from the agar plate was inoculated into 5 ml of NKG broth at 30°C under shaking conditions at 250 rpm for about 20 h. This culture was then inoculated at 10% (v/v) into 100 ml of seed medium with 10 g/l glucose at 250 rpm and 30°C for about 24 h. Flask‐scale cell cultivation was performed with a 250 ml flask containing 60 ml of the fermentation medium^44^. The seed culture prepared above was inoculated at 10% (v/v) into the fermentation medium supplemented with either 40 g/l xylose or 40 g/l glucose or both at 30°C and 250 rpm.

### Batch fermentation

Batch fermentation of engineered strain S09 was carried out in a 5 l quadruple autoclavable fermenter (Shanghai Bailun Biotechnology Co., Ltd.) preloading 2.4 l of fermentation medium without carbon sources. The seed culture was prepared as described above, and then inoculated at 10% (v/v) into 300 ml of seed medium again to obtain the secondary seed. When the OD_600_ reached approximately 3.5–4.5, the secondary seed culture and 300 ml of carbon source were inoculated into the bioreactor^45^. The culture was maintained at 30°C, with pH controlled at 7.0. Aeration was supplied at 1.0 vvm. Agitation was maintained at 300 rpm for the initial 12 h, and then gradually adjusted to 600 rpm to maintain the dissolved oxygen at an appropriate level^23^.

### Determination of CCM metabolite content

The original strain NXdP and engineered strain S09 were cultured in fermentation medium containing a mixture of glucose and xylose at a ratio representative of the lignocellulosic hydrolysate. The samples of fermentation broth at 24 h (the mid‐exponential growth phase) were collected by dilution and centrifugation at 12,000*g* for 20 min at 4°C. The cell pellet was quickly frozen in liquid nitrogen, and then sent to Shanghai Biotree Biotech Co., Ltd. for the determination of intracellular metabolite content related to CCM.

### Determination of cell density and carbon source concentration

For cell growth measurement, cellular biomass was measured by absorbance at 600 nm (OD_600_) with an ultraviolet spectrophotometer (BioDrop; Biochrom). For the analysis of glucose, the seed sample was diluted to a suitable concentration, followed by filtration using a 0.22 μm filter, and the glucose content was determined using an SBA‐40D biosensor (Institute of Biology, Shandong Province Academy of Sciences). The content of xylose was determined using high‐pressure liquid chromatography (HPLC; Agilent 1100) equipped with a refractive index detector. After dilution and filtration, 20 µl aliquots were injected into a 6.5 mm × 300 mm Waters Sugar‐Pak I column (Waters) at a flow rate of 0.5 ml/min and a column temperature of 85°C. The mobile phase used was 50 mg/l calcium disodium EDTA.

### Determination of CDW and sanxan titer

Sanxan is a soluble polysaccharide secreted extracellularly, which can be separated from the bacterial cells through dilution and centrifugation. A certain weight of fermentation broths was diluted at least 10‐fold with deionized water, and then centrifuged at 12,000*g* for 15 min. The supernatant was transferred into new tubes and the pH was adjusted to 3.0 to precipitate the sanxan. The sanxan precipitation and cell pellet were dried to a constant weight and then weighed to analyze the titer and CDW.

### Measurement of the viscosity of fermentation broth

The viscosity of the fermentation broth was measured using a Discovery HR‐1 hybrid rheometer (TA Instruments) equipped with an aluminum parallel plate geometry (diameter, 40 mm; Peltier plate; 1000 μm). The measurement was conducted at 25°C using the flow model at a constant shear rate of 60 s^‐1^ for 60 s. Each fermentation time point sample was measured in triplicate^23^.

### Statistical analysis

All data were presented as the mean ± standard deviation (SD) from three independent replicates. GraphPad Prism 8.0 software was used for graphing and statistical analysis was performed using Student's *t*‐test.

## AUTHOR CONTRIBUTIONS

**Yue Ming**: Data curation; methodology; writing—original draft; writing—review and editing. **Zhuangzhuang Shi**: Software; validation; writing—review and editing. **Yiting Lin**: Validation. **Jue Li**: Investigation. **Weilong Wang**: Validation. **Wei Sha**: Validation. **Guoqiang Li**: Conceptualization; investigation; methodology. **Mengmeng Wu**: Formal analysis; writing—review and editing. **Ting Ma**: Funding acquisition; project administration; supervision.

## ETHICS STATEMENT

No animal or human research was involved in this study.

## CONFLICT OF INTERESTS

The authors declare no conflict of interests.

## Supporting information

Supporting file 1.

## Data Availability

All data produced or analyzed for this study are included in the published article or supplementary information files, or are available from the corresponding authors upon reasonable request.
