# Supplementary material for: Clarification and reprogramming of glucose and xylose metabolism in Sphingomonas sanxanigenens for efficient sanxan biosynthesis
Source: mLife. 2026 Oct 21;5(5):582–96. doi: 10.1002/mlf2.70110 (PMC13685404; doi:10.1002/mlf2.70110)
Supplement: Supplementary file 1 [file MLF2-5-582-s001.docx]

**Supplementary Information**

Figure S1 Construction and characterization of non-sanxan-producing strain NXdPS. (A) Schematic of genes knockout in engineered strain NXdPS. (B) Phenotypic comparison of strains NXdP and NXdPS cultured on NKG agar. (C) Comparison of sanxan titer and CDW between strains NXdP and NXdPS in shake flask fermentation.

Figure S2 The utilization rate of different transporter-knockout strains under solely glucose or xylose cultivation.

Figure S3 The molecular docking between glucose ligand and transporters (A) 0233, (B) 3653, (C) 5235, and between xylose ligand and transporters (D) 0233, (E) 3653, (F) 5235.

Figure S4 The glucose utilization curve of the strains NXdP, S01, and S02.

Figure S5 Schematic diagram of gene *orf2439* knockdown and analysis of its transcription levels in strains NXdPS (Control) and *2439* KD. (A) Schematic diagram of gene *orf2439* knockdown. (B) The relative transcript level of gene *orf2439* in strains Control and *2439* KD.

Figure S6 ^13^C-labeled glucose tracers reveal parallel glycolytic fluxes. (A) GC-MS spectrum of serine. (B) Predictions of metabolite labeling. ^13^C-labeled carbons are colored red; ^12^C-carbons are colored white.

Figure S7 The sanxan titer of strains S08, *0233* OE, *3653* OE, and *5235* OE in shake flask fermentation.

**Table S1. Expression Levels (FPKM) of predicted transporters under different carbon sources (Glc, Xyl, and Glc-Xyl).**

| Gene_id | Glc (FPKM) | Xyl (FPKM) | Glc-Xyl (FPKM) | Predicted function |
| --- | --- | --- | --- | --- |
| *orf0036* | 14.19 | 14.50 | 14.99 | ABC transporter |
| *orf0059* | 28.07 | 25.71 | 16.41 | Phosphate transporter |
| *orf0083* | 0.00 | 0.00 | 0.59 | Sugar transporter |
| *orf0112* | 1.16 | 2.04 | 1.26 | Sugar transporter |
| *orf0228* | 8.91 | 470.66 | 286.55 | Putative MFS permease |
| *orf0233* | 147.62 | 275.81 | 229.28 | Bacterial glucose/galactose transporter |
| *orf0240* | 48.86 | 61.11 | 57.15 | Putative ABC transporter |
| *orf0259* | 7.60 | 10.55 | 6.56 | Putative ABC transporter |
| *orf0289* | 1.24 | 1.24 | 0.89 | Major facilitator transporter |
| *orf0299* | 28.88 | 37.16 | 81.05 | Efflux transporter |
| *orf0325* | 22.88 | 16.16 | 18.17 | Mg^2+^ transporter protein |
| *orf0326* | 16.77 | 18.68 | 17.51 | Phosphate transporter |
| *orf0358* | 274.57 | 322.43 | 241.97 | ABC transporter ATPase subunit |
| *orf0359* | 12.47 | 6.13 | 2.26 | ABC transporter |
| *orf0379* | 3.79 | 5.67 | 4.12 | Formate/nitrite transporter |
| *orf0413* | 30.12 | 33.84 | 38.06 | ABC transporter |
| *orf0414* | 10.21 | 12.71 | 10.53 | ABC transporter |
| *orf0415* | 8.79 | 14.42 | 10.88 | ABC transporter permease protein |
| *orf0421* | 115.57 | 211.57 | 162.91 | ABC transporter-like protein |
| *orf0430* | 4.97 | 3.35 | 4.31 | Putative MFS transporter |
| *orf0446* | 4.91 | 4.55 | 5.34 | ABC-2 type transporter |
| *orf0485* | 1.31 | 2.62 | 1.24 | MFS glycerol uptake transporter |
| *orf0492* | 25.23 | 25.04 | 32.76 | ABC-2 type transporter |
| *orf0493* | 39.80 | 50.53 | 62.52 | Multidrug transporter |
| *orf0500* | 272.59 | 470.24 | 528.84 | ABC transporter |
| *orf0595* | 11.11 | 12.62 | 8.59 | MFS transporter |
| *orf0619* | 95.07 | 84.98 | 77.74 | ABC transporter |
| *orf0667* | 13.42 | 8.38 | 10.50 | Sulfate transporter |
| *orf0723* | 22.24 | 17.69 | 18.37 | MscS family transporter |
| *orf0835* | 3.23 | 3.97 | 3.03 | MFS transporter |
| *orf0846* | 453.32 | 470.92 | 400.23 | Putative ABC transporter |
| *orf0856* | 27.31 | 28.34 | 13.66 | ABC transporter |
| *orf0872* | 3.35 | 2.41 | 4.60 | Transporter |
| *orf0885* | 4.47 | 3.40 | 2.43 | MFS transporter |
| *orf0953* | 11.66 | 7.29 | 10.40 | MFS transporter |
| *orf1013* | 22.63 | 23.80 | 22.32 | Potassium uptake protein |
| *orf1111* | 2.23 | 2.22 | 1.21 | MFS transporter |
| *orf1139* | 2.42 | 4.27 | 2.03 | Putative MFS permease |
| *orf1158* | 3.91 | 5.54 | 3.02 | ABC Fe^3+^ siderophore transporter |
| *orf1160* | 3.11 | 3.41 | 2.59 | Probable citrate transporter |
| *orf1274* | 16.06 | 5.61 | 12.99 | Urea ABC transporter |
| *orf1281* | 55.34 | 158.93 | 120.20 | Putative sugar transporter |
| *orf1343* | 619.94 | 72.53 | 369.73 | Sugar transporter |
| *orf1361* | 23.68 | 11.73 | 10.74 | Hexuronate transporter |
| *orf1415* | 4.01 | 6.68 | 4.46 | Major facilitator transporter |
| *orf1542* | 135.02 | 56.36 | 166.57 | MFS transporter |
| *orf1649* | 68.02 | 48.57 | 51.87 | General substrate transporter |
| *orf1678* | 126. 87 | 111.29 | 144.48 | ABC transporter |
| *orf1688* | 221.01 | 190.60 | 219.42 | ABC transporter |
| *orf1718* | 101.88 | 91.49 | 95.47 | Sulfate transporter |
| *orf1853* | 16.12 | 13.50 | 11.63 | MFS transporter |
| *orf1855* | 29.28 | 26.31 | 22.23 | Major facilitator transporter |
| *orf1897* | 77.45 | 64.89 | 72.47 | Putative MFS permease |
| *orf1941* | 41.30 | 38.81 | 37.75 | Major facilitator transporter |
| *orf2131* | 53.86 | 36.50 | 43.60 | MFS transporter |
| *orf2228* | 0.55 | 0.46 | 0.96 | MFS transporter |
| *orf2314* | 18.86 | 27.02 | 15.03 | Sugar transporter |
| *orf2540* | 86.44 | 65.75 | 91.99 | Magnesium and cobalt transporter |
| *orf2607* | 68.57 | 66.42 | 53.56 | General substrate transporter |
| *orf2735* | 1.87 | 2.32 | 4.31 | Major facilitator transporter |
| *orf2871* | 43.72 | 23.91 | 36.01 | Ammonium transporter |
| *orf2948* | 4.96 | 3.46 | 4.45 | MFS transporter |
| *orf3008* | 1.27 | 2.44 | 1.38 | MFS transporter |
| *orf3129* | 5.93 | 4.73 | 6.09 | Major facilitator transporter |
| *orf3137* | 3.77 | 3.38 | 3.07 | Major facilitator transporter |
| *orf3161* | 13.48 | 11.52 | 9.57 | Sugar transporter |
| *orf3186* | 3.90 | 3.29 | 3.04 | Sugar transporter |
| *orf3196* | 14.72 | 16.39 | 11.99 | MFS transporter |
| *orf3259* | 111.92 | 921.23 | 521.19 | Sugar transporter |
| *orf3353* | 3.74 | 4.07 | 2.99 | Chromate transporter |
| *orf3653* | 11.91 | 10.13 | 10.28 | MFS/sugar transporter |
| *orf3694* | 4.87 | 5.60 | 4.96 | D-galactonate transporter |
| *orf3724* | 2.08 | 11.17 | 5.64 | Ribose ABC transporter |
| *orf3819* | 303.87 | 410.36 | 447.23 | Outer membrane autotransporter |
| *orf3865* | 237.87 | 301.92 | 216.87 | Putative efflux protein |
| *orf4092* | 1.56 | 2.22 | 1.38 | MFS/sugar transporter |
| *orf4151* | 4.51 | 4.14 | 4.90 | MFS permease |
| *orf4169* | 25.52 | 22.49 | 18.17 | MFS/sugar transporter |
| *orf4641* | 4.41 | 3.97 | 3.15 | Autotransporter |
| *orf4994* | 31.07 | 29.09 | 31.96 | Cation diffusion facilitator family transporter |
| *orf5207* | 3.20 | 2.26 | 2.91 | Major facilitator transporter |
| *orf5235* | 1420.72 | 1304.44 | 1164.48 | General substrate transporter |
| *orf5266* | 47.18 | 36.26 | 45.65 | Sugar (and other) transporter |

Note: The values shown in the Table represent the mean of three replicate experiments; error bars are not displayed for the sake of clarity.

**Table S2. The relevant genes that may encode sugar transporters and their descriptions based on the transcriptome.**

| **Gene_id** | **Gene_length (bp)** | **E-value (Blastp against *xylE*/*galP*)** | **Description** |
| --- | --- | --- | --- |
| *orf0083* | 1245 | － | Sugar transporter, glycoside-pentoside-hexuronide (GPH) |
| *orf0228* | 1323 | － | Putative MFS permease |
| *orf0233* | 1326 | － | Bacterial glucose/galactose transporter |
| *orf0835* | 1554 | 1e-87/3e-69 | D-xylose-proton symporter |
| *orf1281* | 975 | － | Putative sugar transporter |
| *orf1343* | 1326 | 5e-89/3e-61 | Sugar transporter |
| *orf2314* | 999 | － | Sugar transporter |
| *orf3008* | 1170 | － | MFS/sugar transport protein |
| *orf3259* | 1422 | 2e-89/5e-61 | Glucose transport protein; Sugar (and other) transporter |
| *orf3653* | 1332 | － | Major facilitator superfamily protein |
| *orf3724* | 1044 | － | Ribose import permease protein RbsC |
| *orf4092* | 1389 | － | MFS/sugar transport |
| *orf5235* | 1326 | － | General substrate transporter |

**Table S3. The viscosity of fermentation broth** **of strains NXdP and S09 grown on co-substrate at different time intervals in 5 L fermenters.**

| **Time** | **Strains** | **Viscosity (Pa.s)** |
| --- | --- | --- |
| 12 h | NXdP | 0.01 ± 0.00 |
|  | S09 | 0.01 ± 0.00 |
| 18 h | NXdP | 0.10 ± 0.00 |
|  | S09 | 0.11 ± 0.01 |
| 24 h | NXdP | 0.27 ± 0.01 |
|  | S09 | 0.29 ± 0.01 |
| 36 h | NXdP | 0.70 ± 0.02 |
|  | S09 | 0.77 ± 0.05 |
| 42 h | NXdP | 0.90 ± 0.04 |
|  | S09 | 0.98 ± 0.07 |
| 48 h | NXdP | 1.03 ± 0.06 |
|  | S09 | 1.12 ± 0.07 |
| 60 h | NXdP | 1.20 ± 0.05 |
|  | S09 | 1.25 ± 0.10 |
| 66 h | NXdP | 1.29 ± 0.10 |
|  | S09 | 1.35 ± 0.08 |
| 72 h | NXdP | 1.31 ± 0.06 |
|  | S09 | 1.40 ± 0.09 |
| 84 h | NXdP | 1.46 ± 0.05 |
|  | S09 | 1.54 ± 0.09 |

**Table S4.** **The bacterial strains and plasmids used in this work**

| **Plasmids and strains** | **Description** | **Source/Reference** |
| --- | --- | --- |
| **Plasmids** |  |  |
| pLO3 | Suicide vector, *sacB*, tet^R^ | (1) |
| pBT | Broad host range vector derived from pBBR1mcs-2 by replacing the promoter *P_lac_* with *P_tac_*, kan^R^ | (2) |
| pLO3-Δ*ssH* | Plasmid for knockout of the native *ssH* gene | This work |
| pLO3-Δ*ssCDG* | Plasmid for knockout of the native *ssCDG* gene | This work |
| pLO3-Δ*0598* | Plasmid for knockout of the native *orf0598* gene | This work |
| pLO3-Δ*2434* | Plasmid for knockout of the native *orf2434* gene | This work |
| pLO3-*P_1281_*-*2439* | Plasmid for integration of *orf2439* gene expressed by the weak promoter *P_1281_* in the *orf5016* site of genome | This work |
| pLO3-Δ*1286* | Plasmid for knockout of the native *orf1286* gene | This work |
| pLO3-Δ*1287* | Plasmid for knockout of the native *orf1287* gene | This work |
| pLO3-Δ*3261* | Plasmid for knockout of the native *orf3261* gene | This work |
| pLO3-Δ*3262* | Plasmid for knockout of the native *orf3262* gene | This work |
| pLO3-Δ*3841* | Plasmid for knockout of the native *orf3841* gene | This work |
| pLO3-Δ*0233* | Plasmid for knockout of the native *orf0233* gene | This work |
| pLO3-Δ*3653* | Plasmid for knockout of the native *orf3653* gene | This work |
| pLO3-Δ*5235* | Plasmid for knockout of the native *orf5235* gene | This work |
| pLO3-Δ*GIO* | Plasmid for knockout of the native *orf0649-0656* gene | This work |
| pLO3-Δ*1342* | Plasmid for knockout of the native *orf1342* gene | This work |
| pBT-*0598* | Plasmid for expressing *orf0598* gene | This work |
| pBT-*2434* | Plasmid for expressing *orf2434* gene | This work |
| pBT-*0233* | Plasmid for expressing *orf0233* gene | This work |
| pBT-*3653* | Plasmid for expressing *orf3653* gene | This work |
| pBT-*5235* | Plasmid for expressing *orf5235* gene | This work |
| **Strains** |  |  |
| *Escherichia coli* S17 | Strain used for biparental conjugation, *RecA thi pro hsdR^−^* M^+^ RP4, Sm^R^ Amp^R^ Kan^R^ | This work |
| *Sphingomonas sanxanigenens* NXdP | PHB-defective strain from the wild-type strain NX02, PHB^−^ | This work |
| *Sphingomonas sanxanigenens* NXdPS | Engineered strain, PHB^−^, Sanxan^−^ | This work |
| Δ*0598* | NXdPS derivate, knockout of the native *orf0598* gene | This work |
| Δ*4389* | NXdPS derivate, knockout of the native *orf4389* gene | This work |
| Δ*2434* | NXdPS derivate, knockout of the native *orf2434* gene | This work |
| *2439* KD | NXdPS derivate, knockout of the native *orf2434* gene with integration of *orf2439* gene expressed by the weak promoter *P_1281_* in the *orf5016* site of genome | This work |
| X1 | NXdPS derivate, knockout of the native *orf3841* gene | This work |
| X2 | NXdPS derivate, knockout of the native *orf3262* gene | This work |
| X3 | NXdPS derivate, knockout of the native *orf1286-1287* gene | This work |
| X4 | NXdPS derivate, knockout of the native *orf3261-3262* gene | This work |
| X5 | NXdPS derivate, knockout of the native *orf1286-1287*, and *orf3262* gene | This work |
| X6 | NXdPS derivate, knockout of the native *orf1286-1287*, and *orf3841* gene | This work |
| X7 | NXdPS derivate, knockout of the native *orf1286-1287*, and *orf3261-3262* gene | This work |
| *0233* KO | NXdPS derivate, knockout of the native *orf0233* gene | This work |
| *0233* CO | Strain *0233* KO harboring the plasmid pBT-*0233* | This work |
| *3653* KO | NXdPS derivate, knockout of the native *orf3653* gene | This work |
| *3653* CO | Strain *3653* KO harboring the plasmid pBT-*3653* | This work |
| *5235* KO | NXdPS derivate, knockout of the native *orf5235* gene | This work |
| *5235* CO | Strain *5235* KO harboring the plasmid pBT-*5235* | This work |
| S01 | NXdP derivate, knockout of the native *orf2434* gene | This work |
| S02 | Strain NXdP harboring the plasmid pBT-*2434* | This work |
| S03 | NXdP derivate, knockout of the native *orf2434* gene with integration of *orf2439* gene expressed by the weak promoter *P_1281_* in the *orf5016* site of genome | This work |
| S04 | NXdP derivate, integration of *orf2439* gene expressed by the weak promoter *P_1281_* in the *orf5016* site of genome | This work |
| S05 | Strain NXdP harboring the plasmid pBT-*0598* | This work |
| S06 | NXdP derivate, knockout of the native *orf0649-0656* gene | This work |
| S07 | NXdP derivate, knockout of the native *orf1342* gene | This work |
| S08 | S06 derivate, knockout of the native *orf1342* gene | This work |
| S09 | S08 derivate, knockout of the native *orf3841* gene | This work |
| S10 | S08 derivate, knockout of the native *orf3261-orf3262* gene | This work |

**References:**

1 Lenz O, Friedrich B. A novel multicomponent regulatory system mediates H_2_ sensing in *Alcaligenes eutrophus*. *Proc Natl Acad Sci U S A*. 1998; **95**: 12474-12479.

2 Wu M, Qu J, Tian X, Zhao X, Shen Y, Shi Z, et al. Tailor-made polysaccharides containing uniformly distributed repeating units based on the xanthan gum skeleton. *Int J Biol Macromol*. 2019; **131**: 646-653.
